# Certified Production and Physicochemical Compliance of Vastedda Della Valle del Belìce PDO: A Five-Year Analysis of Official Certification Data (2021–2025)

**DOI:** 10.3390/foods15213794

**Published:** 2026-10-23

**Authors:** Rosario Maria Catalano, Aldo Migliazzo, Delia Gambino, Giuseppe Barbaccia, Lucia Galuppo, Giuseppe Lo Bue, Antonio D’Amico, Armando Massimo Ceraulo, Francesca Di Gaudio

**Affiliations:** 1 Istituto Zooprofilattico Sperimentale della Sicilia “A. Mirri”, Via G. Marinuzzi, 3, 90129 Palermo, Italy; rosario.catalano@izssicilia.it (R.M.C.); aldo.migliazzo@izssicilia.it (A.M.); giuseppe.barbaccia@izssicilia.it (G.B.); lucia.galuppo@izssicilia.it (L.G.); giuseppe.lobue@izssicilia.it (G.L.B.); antonio.damico@izssicilia.it (A.D.); armando.ceraulo@izssicilia.it (A.M.C.); 2 Department of Agricultural, Food and Forestry Science (SAAF), Università di Palermo, Viale delle Scienze 13, 90128 Palermo, Italy

**Keywords:** Vastedda della Valle del Belìce, PDO cheese, official certification data, physicochemical compliance, food quality monitoring

## Abstract

Vastedda della Valle del Belìce Protected Designation of Origin (PDO) is a traditional Sicilian stretched-curd cheese produced from raw sheep’s milk. This study analysed data generated during routine certification and control activities conducted by the Control Body from 2021 to 2025 to assess compliance with the PDO Production Specification and describe year-to-year variation in certified production and physicochemical parameters. Certified production volumes and 96 analytical reports were retrospectively analysed in aggregated and anonymised form. Mean annual certified production was 26,295 kg. Mean fat content on a fresh-weight basis was 24.14%, with annual means ranging from 23.95% to 24.44%; however, one sample collected in 2021 showed a fat content below the minimum regulatory threshold of 18%. Mean sodium chloride content was 0.54%, well below the maximum permitted value of 2.7%. Among the 96 analytical reports included in the study, 95 (98.96%) met both physicochemical criteria evaluated in this study (fat and NaCl content), whereas one report was classified as non-compliant because of its fat content. These findings indicate a high observed level of compliance among the analytical reports examined and support the use of official certification data as a complementary resource for monitoring PDO product conformity over time under routine production conditions.

## 1. Introduction

Protected Designations of Origin (PDOs) represent one of the main instruments for safeguarding traditional agri-food products, as they preserve the link between the product, its geographical area of origin, and the production practices that determine its distinctive characteristics [1]. In the dairy sector, the quality of PDO products results from the interaction among environmental factors, animal genetic resources, farming systems, and processing technologies developed and consolidated over time [2]. Such interactions may contribute to variability in the physicochemical characteristics of traditional dairy products, particularly when production relies on raw milk and traditional processing practices.

Vastedda della Valle del Belìce PDO is a traditional Sicilian stretched-curd cheese produced exclusively from whole raw milk obtained from Valle del Belìce sheep reared within a defined production area comprising municipalities in the provinces of Agrigento, Trapani, and Palermo [3]. The cheese is characterized by a distinctive production process that includes curd acidification, stretching in hot water, salting, and subsequent shaping into its characteristic flattened form. The use of raw milk preserves the indigenous microbiota associated with the milk and traditional production environment, which may contribute to the development of the cheese microbiota and its characteristic properties [4,5,6,7]. At the same time, this traditional approach requires strict compliance with the requirements established by the Production Specification and with good hygienic practices throughout all stages of production.

In addition, the biological variability of raw ewe’s milk may contribute to variation in the physicochemical characteristics of the cheese. Previous research has shown that the characteristics of Vastedda della Valle del Belìce are influenced by raw-milk composition, seasonal and environmental conditions, indigenous microbiota, and cheesemaking practices [5,8,9]. Such variability is particularly relevant in PDO products, where traditional production practices must coexist with predefined requirements for product identity and regulatory conformity [10]. Consequently, the assessment of physicochemical parameters under routine production conditions represents an important complement to studies conducted under controlled experimental conditions.

Compliance of Vastedda della Valle del Belìce PDO with the requirements established by the Production Specification is verified through the certification system entrusted to the Istituto Zooprofilattico Sperimentale della Sicilia “A. Mirri” (IZS Sicilia), acting as the Control Body (CB) authorized by the Ministry of Agriculture, Food Sovereignty and Forestry (MASAF) [11]. Within this framework, documentary, inspection, and analytical controls are carried out according to the procedures established for the PDO certification system, generating a database containing technical, production, and analytical information collected according to standardized procedures. Unlike experimental studies conducted under controlled conditions, official certification records reflect products sampled during routine commercial activities. Their analysis over consecutive years can provide complementary information on certified production volumes and physicochemical conformity.

Although the scientific literature has extensively described the microbiological, technological, and qualitative aspects of Vastedda della Valle del Belìce PDO, previous studies have generally focused on specific aspects of the product, whereas information derived from the systematic analysis of official certification data collected over consecutive production years remains limited. To the best of the authors’ knowledge, no previous study has systematically analysed five consecutive years of official certification data for Vastedda della Valle del Belìce PDO to characterize certified production and physicochemical compliance under routine commercial production conditions. The aim of this study was therefore twofold: to assess compliance with the physicochemical requirements established by the PDO Production Specification and to describe year-to-year variation in certified production volumes and physicochemical parameters recorded over the five-year period (2021–2025). The findings refer to the available certification records and are not intended to represent a comprehensive assessment of the entire PDO production during the study period.

## 2. Materials and Methods

### 2.1. Study Design

A retrospective observational study was conducted using data generated during routine certification and control activities performed by the designated Control Body (CB) for Vastedda della Valle del Belìce PDO cheese. The study included inspections carried out between 1 January 2021 and 31 December 2025 under the PDO certification and control system. The study was based on anonymized certification records collected for regulatory purposes, and involved no experimental intervention. It therefore represents a retrospective observational analysis of routine data generated under commercial production conditions.

### 2.2. Study Setting and PDO Production System

Vastedda della Valle del Belìce PDO cheese is produced according to the Production Specification approved by the MASAF, which defines the geographical production area, animal husbandry requirements, cheesemaking procedures, and traceability system governing the entire production chain [3]. The PDO production area comprises 18 municipalities located in the provinces of Agrigento, Trapani, and Palermo (Sicily, Italy).

Milk is obtained exclusively from Valle del Belìce sheep reared within the designated geographical area under predominantly extensive farming systems. Animal feeding is mainly based on natural grazing and locally produced forages, while the Production Specification prohibits the use of genetically modified organisms (GMOs), animal-derived by-products, and selected feed, including plants or seeds of Trigonella, tapioca, and cassava.

The traditional manufacturing process requires the use of traditional copper and wooden equipment. The use of wooden surfaces has been associated with the maintenance of resident autochthonous microbial communities characteristic of traditional dairy production [12,13,14]. The whole raw ewe’s milk must be processed within 48 h after the first milking. The milk is heated to 36–40 °C and coagulated using lamb rennet in paste form in tinned-copper vats. The resulting curd is then broken in traditional wooden vats and allowed to mature in rush baskets (fuscelle) for 24–48 h.

Wooden equipment, particularly the vat used for curd breaking and the “piddiaturi”, a traditional wooden implement used to cut the cheese mass during cheesemaking, can harbor resident microbial communities, predominantly composed of autochthonous lactic acid bacteria. These microorganisms contribute to curd acidification and to the achievement of the pH values (4.7–5.5) specified for the PDO product [7,9]. The curd is subsequently immersed in water at 80–90 °C for manual stretching, then portioned and manually shaped in ceramic deep plates. After cooling and firming for 6–12 h, the cheese forms are immersed in saturated brine at room temperature for 30 min to 2 h, as specified in the PDO Production Specification. The Production Specification establishes a commercial shelf life of 90 days and defines specific physicochemical and dimensional requirements for the certified product [3].

### 2.3. Certification and Control System

Compliance with the Production Specification is verified through the official certification system managed by the IZS Sicilia, acting as the Control Body authorized by MASAF.

Certification and control activities include documentary verification, on-site inspections, traceability assessment, and physicochemical analyses performed throughout the production chain according to the Control Plan. Documentary inspections are aimed primarily at verifying the traceability and compliance of the production chain, whereas analytical inspections include the sampling of selected production lots and laboratory determinations. These activities generate a standardized certification database containing production, traceability, and laboratory information collected under harmonized regulatory procedures.

The certification database served as the primary data source for this study. Only variables routinely available and consistently recorded throughout the study period were considered.

Compliance was assessed against the Vastedda della Valle del Belìce PDO Production Specification [3], which establishes physicochemical, dimensional, morphological, and sensory requirements for the cheese. The present study focused exclusively on fat and sodium chloride (NaCl) content, as these were the physicochemical parameters consistently available in the certification records throughout the study period. The Specification sets minimum fat contents of 18% on a fresh-weight basis and 35% on a dry-matter basis, and maximum NaCl contents of 2.7% on a fresh-weight basis and 5% on a dry-matter basis. Only the fresh-weight thresholds were applied in this study (fat ≥ 18%; NaCl ≤ 2.7%). An analytical report was classified as compliant with respect to the parameters assessed only when both thresholds were met; failure to meet either threshold resulted in a non-compliant classification. The assessment was therefore restricted to fat and NaCl content and did not cover all requirements established by the PDO Production Specification.

For international context, the Codex Alimentarius General Standard for Cheese (CXS 283-1978) provides general provisions for cheese, including milkfat classification based on fat in dry matter (FDM) [15]. However, these provisions do not establish directly equivalent criteria for the fresh-weight fat and NaCl thresholds specified in the Vastedda della Valle del Belìce PDO Production Specification. Therefore, the Codex Standard was considered as an international reference, but no direct numerical comparison was performed.

### 2.4. Sampling and Analytical Determinations

Only analytical inspections performed between 2021 and 2025 under the Control Plan were included in the present study. Documentary inspections that did not involve laboratory analyses were excluded.

According to the Control Plan, the Control Body performs physical and analytical checks on finished cheese to verify compliance with product specification. The number of cheese forms sampled from each selected lot depends on lot size: one form from lots comprising 1–100 forms, two forms from lots comprising 101–200 forms, and three forms from lots comprising more than 200 forms. All selected lots included in the present study comprised no more than 100 forms; therefore, one cheese form was collected at each sampling session. Each sampled cheese form was divided into four equivalent aliquots, identified by Roman numerals I–IV. Aliquots I and II were submitted to the IZS Sicilia laboratories. Aliquot I was used for the scheduled analyses, whereas aliquot II was retained at the laboratory for possible repeat or confirmatory analyses in the event of a dispute concerning the analytical results. Aliquot III was retained at the dairy plant and made available to the operator, and aliquot IV was stored at the Control Body’s premises for possible confirmatory or review analysis in the event of a dispute concerning the analytical results.

A total of 96 analytical reports were included in the study, corresponding to 96 inspection visits involving sampling conducted by the Control Body during the 2021–2025 period. Each report corresponded to a distinct production lot, resulting in 96 distinct lots sampled during official control activities. Only the initial analytical results obtained from aliquot I were included in the dataset; no repeated or confirmatory results were included. The dataset was restricted to lots sampled during these inspections and did not necessarily encompass all lots produced or certified during the study period.

Physicochemical determinations were performed at the laboratories of the IZS Sicilia using methods accredited in accordance with UNI CEI EN ISO/IEC 17025:2018 [16]. The parameters analysed were fat content and sodium chloride (NaCl) content, both expressed as percentages of fresh weight. Fat and NaCl contents were determined by near-infrared (NIR) spectroscopy using a FoodScan analyser (FOSS, Denmark), with reference to ISO 21543:2020|IDF 201:2020, which describes the application of near-infrared spectroscopy to milk products [17]. The analytical method and instrumentation remained unchanged throughout the study period (2021–2025).

### 2.5. Data Collection and Anonymisation

The study included all seven dairies participating in the Vastedda della Valle del Belìce PDO certification system during the study period. All seven dairies were represented in the analytical dataset, although the number of sampled lots varied among producers and years. In particular, Company G did not produce Vastedda della Valle del Belìce PDO in 2025, as formally communicated to the Control Body. Samples were collected from production lots available at the dairy at the time of inspection; therefore, the number of samples collected from each dairy was determined by routine control activities rather than by a balanced sampling design. The 96 analytical reports corresponded to 96 distinct production lots sampled across the seven dairies. The dataset did not necessarily encompass all lots produced or certified during the study period (Table 1).

Production volumes and analytical results were extracted from the certification database maintained by IZS Sicilia. To ensure confidentiality, each dairy plant was assigned a unique anonymous alphabetical code (Companies A–G). Only aggregated and anonymised data were used throughout the study, and no information allowing the identification of individual producers was included in the analyses or reported in the results.

The database was checked for completeness, consistency of measurement units, duplicate records, and missing values before statistical analysis. Records with missing analytical results were excluded only from analyses involving the corresponding variable.

### 2.6. Statistical Analyses

The 96 analytical reports included in the study did not originate from an experimental study design but represented all analytical samples collected from production lots selected by the Control Body during the 2021–2025 observation period.

Given the inspection-based nature of the dataset, the statistical analysis was limited to descriptive statistics, with analytical results aggregated by calendar year. For the physicochemical parameters included in the Control Plan, namely fat content on a fresh-weight basis and sodium chloride content on a fresh-weight basis, the arithmetic mean, standard deviation (SD), and observed range (minimum–maximum) were calculated for each year.

This descriptive approach was considered appropriate for the exploratory and descriptive aim of the study, which was to characterize physicochemical compliance and temporal variability among samples collected under routine commercial production conditions. No inferential comparisons between years were performed because the data originated from inspection-based certification activities rather than from an a priori experimental or probabilistic sampling design.

## 3. Results

### 3.1. Certified Production and Production Structure

During the 2021–2025 observation period, the total certified production of Vastedda della Valle del Belìce PDO amounted to 131,474.9 kg. Certified production volumes showed moderate year-to-year variation over the five-year period, without a consistent increasing or decreasing pattern (Table 2).

The distribution of production among certified producers showed a clearly heterogeneous structure, characterized by a small number of high-capacity dairies alongside several smaller artisanal producers. In particular, four companies (D, B, A, and C) accounted collectively for 86.23% of the total certified production (Figure 1), while the remaining producers contributed a minor share of the overall output. Company G did not produce Vastedda della Valle del Belìce PDO in 2025, as formally communicated to the Control Body.

According to official production records, 234,777 cheese units were produced over the five-year period, corresponding to a total certified production volume of 131,474.90 kg. Records from official controls indicated that the cheese units collected during control activities complied with the 0.50–0.70 kg weight range specified in the PDO Production Specification [3].

### 3.2. Physicochemical Characteristics and Compliance

The Production Specification establishes specific physicochemical requirements for Vastedda della Valle del Belìce PDO, including a minimum fat content of 18% and a maximum sodium chloride content of 2.7%, both expressed on a fresh-weight basis [3].

A total of 96 analytical reports were included in the analysis. Among these, 95 (98.96%) met both physicochemical criteria evaluated in this study (fat and NaCl content), whereas one report (1.04%) was classified as non-compliant.

Annual descriptive statistics for fat and sodium chloride content are reported in Table 3.

Mean fat content ranged from 23.95 ± 1.78% in 2025 to 24.44 ± 2.16% in 2023, while sodium chloride content showed annual mean values ranging from 0.48 ± 0.13% in 2023 to 0.59 ± 0.14% in 2024 and 2025. Fat content was above the minimum regulatory threshold in all but one sample. In 2021, a single sample showed a fat content of 15.93%, below the minimum limit of 18%. This represented the only non-compliant determination for fat content during the study period. The lot concerned was subjected to the measures established under the Control Plan, including product downgrading.

Sodium chloride content remained below the maximum regulatory limit in all samples.

Annual variation in fat and sodium chloride content is shown in Figure 2.

## 4. Discussion

The present study provides a five-year assessment of compliance with the fat and NaCl requirements of Vastedda della Valle del Belìce PDO, based on official certification data. Among the 96 analytical reports included in the study, 95 (98.96%) met the two physicochemical requirements assessed according to the PDO Production Specification [3]. These findings indicate a high observed level of compliance for fat and NaCl content among the samples analysed under routine commercial production conditions. However, the results describe the analytical reports included in the dataset and should not be interpreted as an estimate of compliance across the entire PDO production during the study period.

Fat content showed relatively consistent annual mean values, ranging from 23.95% to 24.44%, and remained substantially above the minimum regulatory threshold of 18% in all years except for one isolated sample collected in 2021, which showed a fat content of 15.93%. The absence of further non-compliant results in the subsequent four years provides no evidence of a recurrent deviation among the monitored samples. The composition of ewe’s milk may vary according to several interacting factors, including stage of lactation, nutritional status, season, environmental conditions, and management practices [18,19]. In Mediterranean production systems, environmental conditions and pasture availability may also affect milk composition [8,18,20,21]. However, these factors could not be directly evaluated in the present study because corresponding animal-level, feeding, climatic, and pre-processing milk-composition data were not available. Importantly, the isolated non-compliant lot was identified through the official certification procedures and managed according to the corrective measures established by the Control Plan.

Sodium chloride content showed even lower interannual variability, with annual mean values ranging from 0.48% to 0.59%, and remained well below the maximum regulatory limit of 2.7% throughout the study period. No samples exceeded the maximum permitted NaCl content. Salt concentration is an important technological characteristic of cheese, contributing to flavour development and influencing water activity and microbial growth [22,23]. The relatively limited variation in annual mean values observed in the present study suggests overall consistency in this physicochemical characteristic among the samples collected during the study period. Nevertheless, process-level variables such as brine concentration, temperature, immersion time, and salt diffusion kinetics were not available and therefore could not be assessed. Although salt concentration can influence water activity and microbial growth, the available data did not allow the relationship between NaCl content and microbiological stability throughout the shelf-life to be assessed. Future studies should integrate physicochemical and microbiological analyses at defined stages of storage to investigate this relationship.

The observed physicochemical consistency should also be interpreted in the context of the traditional manufacturing process of Vastedda, which includes spontaneous curd acidification, high-temperature stretching, and brining. These technological steps contribute to the distinctive characteristics of the product and have been extensively described in previous studies [5,12,13,24,25]. However, because the present study did not include microbiological or detailed process-level measurements, it was not possible to evaluate the specific contribution of individual manufacturing steps to the physicochemical characteristics observed.

An important contribution of the present study is the use of routinely generated official certification data as a research resource to assess certified production and physicochemical compliance over five consecutive production years. Previous studies on Vastedda have investigated its technological and compositional characteristics under specific sampling conditions [5,8,12,26]. Mucchetti et al. [7] reported a mean fat content of 22.4 ± 3.70% and a mean NaCl content of 0.47 ± 0.27% in 20 cheese samples, values broadly comparable to those observed in the present study (24.14% and 0.54%, respectively). Todaro et al. [8], who analysed 18 samples from six dairies across three production seasons, found that fat content expressed on a dry-matter basis differed significantly by season, whereas salt content did not. Direct quantitative comparisons with the latter study are limited by the different basis of expression. The present analysis complements this evidence by describing physicochemical compliance under routine commercial production conditions. Such records may therefore provide a useful complementary source of information for monitoring compliance with predefined PDO requirements over time.

Several limitations should be considered. The observed 98.96% compliance represents the proportion of compliant analytical reports included in the study and should not be interpreted as an unbiased estimate of compliance across all Vastedda della Valle del Belìce produced during 2021–2025, because sampling was determined by the routine Control Plan rather than by an a priori probabilistic sampling design. Although the 96 official analytical reports provide a useful overview of routine control activities, their number and uneven annual distribution limit the robustness of the assessment of temporal variability. Each analytical report corresponded to a distinct production lot identified in the certification database. The retrospective nature of the dataset restricted the analysis to variables routinely collected within the certification system. Consequently, potential determinants of physicochemical variability, including ewe-level characteristics, lactation stage, diet, climatic conditions, milk composition before processing, and detailed cheesemaking parameters, could not be evaluated. Therefore, the observed variations cannot be attributed to specific biological, climatic, or technological factors. The descriptive approach also precluded causal inference and formal testing of temporal or site-specific differences. In addition, certified production was unevenly distributed among dairies, with four producers accounting for most of the total output. Therefore, the observed compliance profile should not be interpreted as equally representative of producers with different production volumes or as a production-weighted estimate of the PDO sector. Finally, the five-year observation period provides a description of the monitored years but does not establish whether the observed values differ from longer-term historical patterns.

## 5. Conclusions

The present five-year analysis of official certification data showed a high observed level of compliance with the fat and NaCl requirements of the Vastedda della Valle del Belìce PDO, with 95 of 96 analytical reports (98.96%) meeting both physicochemical criteria evaluated in this study. The monitored physicochemical characteristics showed overall consistency across the study period, with NaCl content consistently within the prescribed limit and only one isolated non-compliant result for fat content. These findings support the value of routinely generated certification data as a complementary resource for monitoring compliance with specific PDO requirements over time. Future research should build on standardized longitudinal datasets and link physicochemical results with information on season, milk composition, animal diet, stage of lactation, and production and maturation conditions. The inclusion of microbiological assessments throughout the shelf-life and other relevant product-quality criteria would also help clarify the relationship between physicochemical characteristics and product stability.

## Figures and Tables

**Figure 1:**
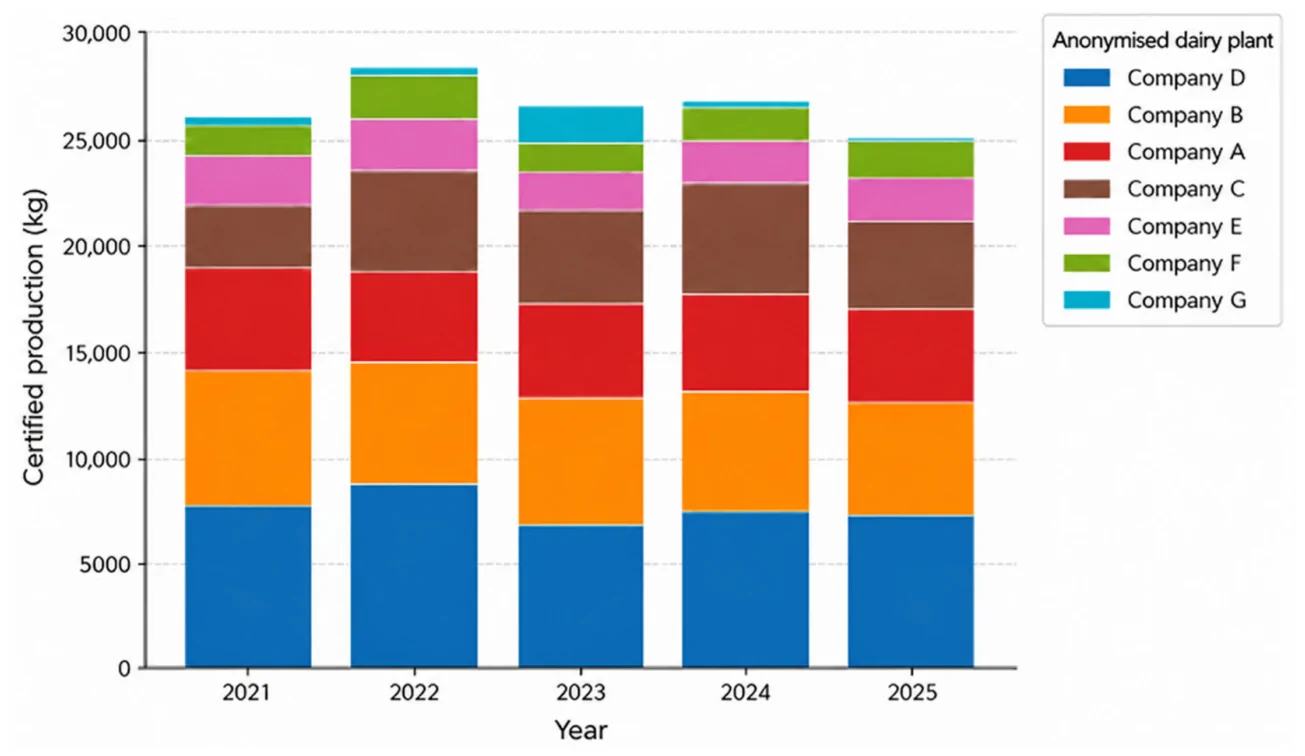
Annual certified production volumes of Vastedda della Valle del Belìce PDO cheese by anonymised dairy plant, 2021–2025.

**Figure 2:**
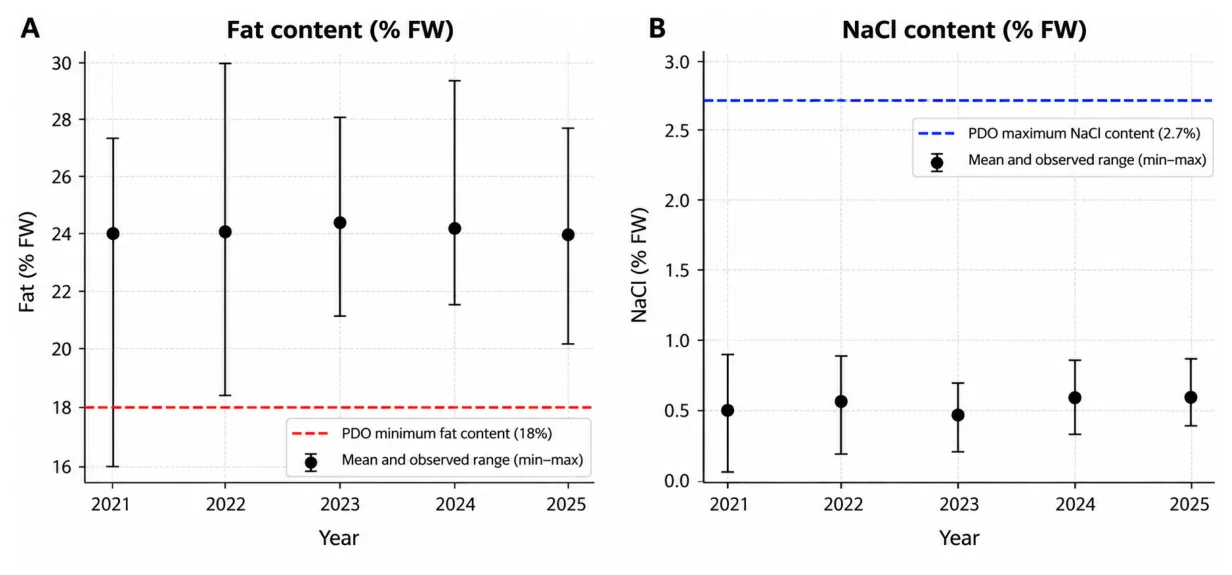
Annual variation in fat (**A**) and NaCl (**B**) content on a fresh-weight basis (FW), 2021–2025. Black markers indicate the annual arithmetic mean, and error bars represent the observed minimum–maximum range. Horizontal dotted lines indicate the limits specified in the PDO Production Specification: a minimum fat content of 18% and a maximum NaCl content of 2.7%.

**Table 1:** Annual distribution of analysed production batches by producer, 2021–2025.

Companies	2021	2022	2023	2024	2025	Total Batches Analysed
A	5	5	3	3	3	19
B	6	4	3	3	3	19
C	3	4	3	3	3	16
D	3	3	1	1	2	10
E	5	5	4	2	3	19
F	2	3	1	2	1	9
G	1	1	1	1	0	4
Total	25	25	16	15	15	96

**Table 2:** Annual certified production (kg) of Vastedda della Valle del Belìce PDO cheese by anonymised dairy plant during the study period (2021–2025).

Anonymised Dairy Plant	2021	2022	2023	2024	2025	Total
Company A	5954.5	4998.6	6196.0	5460.0	5412.4	28,021.5
Company B	6370.0	6056.0	6066.0	5620.0	5316.0	29,428.0
Company C	2531.0	4717.0	4987.0	6043.0	5032.6	23,310.6
Company D	7411.0	8004.0	4864.0	6530.0	5810.0	32,619.0
Company E	2054.0	1872.0	1622.2	1455.0	1623.0	8626.2
Company F	1217.6	2049.2	1240.8	1384.7	1738.6	7630.9
Company G	226.2	156.1	1417.5	38.9	0.0	1838.7
Total certified production	25,764.3	27,852.9	26,393.5	26,531.6	24,932.6	131,474.9

Note: Production volumes reported for each anonymised dairy plant include any changes in company ownership or legal status recorded by the Control Body during the study period, provided that production continued at the same manufacturing site.

**Table 3:** Descriptive statistics of physicochemical parameters determined in samples of Vastedda della Valle del Belìce PDO cheese, 2021–2025.

Year	n	Fat (% FW), Mean ± SD	Fat Range (%)	NaCl (% FW), Mean ± SD	NaCl (%) Range
2021	25	24.01 ± 2.33	15.93–27.29	0.50 ± 0.19	0.03–0.89
2022	25	24.12 ± 3.16	18.26–29.99	0.56 ± 0.18	0.20–0.88
2023	16	24.44 ± 2.16	21.23–28.09	0.48 ± 0.13	0.21–0.67
2024	15	24.24 ± 2.08	21.51–29.42	0.59 ± 0.14	0.33–0.85
2025	15	23.95 ± 1.78	20.13–27.70	0.59 ± 0.14	0.41–0.88

Values are expressed as mean ± standard deviation (SD). Range indicates minimum–maximum values. Fat and NaCl contents are expressed as percentages of fresh weight. n = number of analytical samples.

## Data Availability

The datasets analysed during the current study are not publicly available because they were derived from institutional certification activities and are subject to confidentiality requirements. Aggregated data supporting the findings of this study are available from the corresponding author upon reasonable request, subject to applicable institutional and confidentiality requirements.
